# Transcriptome Analysis of Epithelioma Papulosum Cyprini Cells Infected by Reovirus Isolated from Allogynogenetic Silver Crucian Carp

**DOI:** 10.3390/v10030135

**Published:** 2018-03-18

**Authors:** Cui-Yu Ba, Xiao-Yan Du, Pei-Jun Zhang, Ping Chen, Ya-Nan Cai, Yue-Hong Li

**Affiliations:** 1 Animal Science and Technology, Jilin Agriculture University, Changchun 130118, China; bacuiyu@163.com (C.-Y.B.);Caiyanan0925@163.com (Y.-N.C.); 2 College of Life Sciences, Jilin Agriculture University, Changchun 130118, China; 3 Freshwater Fisheries Research Institute of Jilin Province, Changchun 130000, China; dxypa@tom.com; 4 Health Monitoring and Inspection Center of Jilin Province, Changchun 130062, China; zhangpeijun63@sina.com; 5 College of Food Science and Engineering, Jilin Agriculture University, Changchun 130118, China; ccchenping@sina.com

**Keywords:** transcriptome, EPC, reovirus, allogynogenetic silver crucian carp

## Abstract

The present study aimed to identify differentially expressed genes (DEGs) and major signal transduction pathways that were related to the immune response of epithelioma papulosum cyprinid (EPC) cells to reoviruses isolated from allogynogenetic silver crucian carp. The study also lays a theoretical foundation for the pathogenesis and immunity of the reovirus, which is helpful to the breeding of cyprinids fish. Reovirus infected and uninfected EPC cells were analyzed by using a new-generation high-throughput sequencing technology. DEGs were identified, annotated, and classified, and the signal pathways involved in the response to reovirus infection were identified by using bioinformatics tool. The data were assembled into 92,101 contigs with an average length of 835.24 bp and an N50 value of 1432 nt. Differential expression analysis of all the genes identified 3316 DEGs at a false discovery rate (FDR) of <0.01 and a fold-change of ≥3, of which 1691 were upregulated genes, 1625 were downregulated, and about 305 were immune-related genes. Gene Ontology (GO) enrichment analysis resulted in the annotation of 3941 GO terms, including 2719 biological processes (37,810 unigenes), 376 cell components (7943 unigenes), and 846 molecular functions (11,750 unigenes). KEGG metabolic pathway analysis matched the DEGs from pre-and post-infection EPC cells to 193 pathways, of which 35 were immune-related, including the Toll-like receptor, cytokine-cytokine receptor interaction, and the JAK-STAT signaling pathways.

## 1. Introduction

Aquareoviruses are characterized to have a double capsid, icosahedral symmetry, and no capsule. The average diameter of aquareoviruses is 60–70 nm and its genome consists of 11 segments of double-stranded RNA [1,2]. Aquatic reoviruses have infected various economically important economic aquaculture fishes such as grass carp, black carp, barley fish, Atlantic salmon, and rare crucian carp, thereby resulting in higher mortality rates. Besides aquatic animals, reoviruses have been isolated from ostriches, bats, and ducks. However, reports on the isolation of reoviruses that infect allogynogenetic crucian carp are limited.

The transcriptome is the sum of all the transcripts of a given organism in a given state, including not only mRNAs but also non-coding RNAs [3,4]. The transcriptome is an essential link between genetic information and biological function (proteome). New genes and transcripts are often identified by using the latest high-throughput sequencing technology and correlations are calculated via mRNA expression analysis [5,6,7]. Transcriptome analysis is mainly used in studies of differential gene expression, allele-specific expression, alternative splicing, fusion genes and other related studies [8,9,10]. Transcriptome sequencing (RNA-seq) pertains to the direct sequencing of a cDNA sequence using a large-scale sequencing technology, thereby resulting in tens of millions of reads that could determine its transcription level by direct comparison of the number of reads in the genomic region [11]. In recent years, RNA sequencing has become a widely used method for fish-virus interaction studies. Due to our limited understanding of the molecular mechanism underlying reovirus infection in Cyprinidae, most research studies on the resistance to reoviruses have mainly focused on immune-related genes such as IL-10, HSP70, and HSP90, as well as other areas such as antimicrobial peptides (AMPs), proteomics analysis, and physical and chemical control strategies.

To understand the molecular immune mechanism of reoviruses in cyprinid fish, the present study analyzed the transcriptome of epithelioma papulosum cyprinid (EPC) cells that were infected by reovirus using the Illumina/Solexa high-throughput sequencing technique. We investigated the mRNA expression levels, immune-related genes, and pathways, and identified several simple repeat sequences (SSRs) and single nucleotide polymorphisms (SNPs). The findings of the present study may be utilized in the elucidation of the molecular mechanism underlying the potential interaction of reoviruses isolated from allogynogenetic crucian carp with EPCs.

## 2. Materials and Methods

### 2.1. Isolation, Inoculation, and Sampling of Reoviruses from Allogynogenetic Silver Carassius auratus

The allogynogenetic reoviruses used in the present study were isolated from diseased gibel carp by our laboratory in a fishing ground in Jilin Province in 2015. The EPC cells and crucian carp reoviruses were stored at the Jilin Agricultural University aquatic laboratory. The EPC cells were cultured in 75-cm^2^ flasks (BD Biosciences, Franklin Lakes, NJ, USA) with M199 cell culture medium containing 10% fetal bovine serum (Life Technologies, Carlsbad, CA, USA) and 1% double antibody (Sigma-Aldrich, St. Louis, MO, USA) at 27 °C. Upon reaching 80% confluency, the reovirus was added to the cell culture bottle for propagation. Upon reaching a confluency of >80%, the EPCs were harvested and stored at −80 °C as the virus mother liquor. The titer of the virus was measured on a 96-well plate covered with EPC cells using the Karber method.

**Figure 1:**
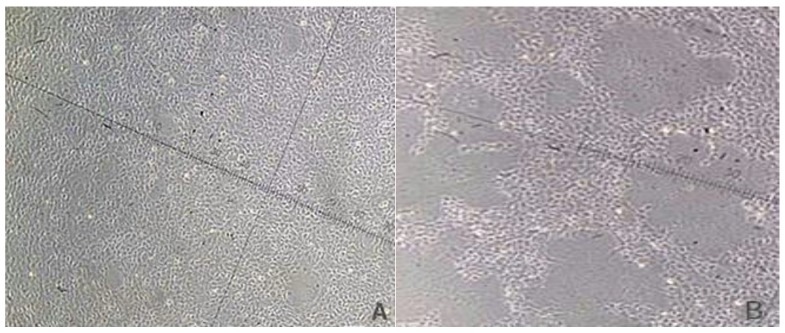
Cytopathy of epithelioma papulosum cyprinid (EPC) cells after virus infection (100×).

Approximately 1.4 × 10^7^ cells were seeded onto 75-cm^2^ culture flasks (BD Biosciences) and 90% confluency was achieved after incubating for 24 h in a 27 °C incubator. Reoviruses with a multiplicity of infection (MOI) of 10 were added to the cell cultures. The same volume of M199 medium was added to a control flask (Figure 1A), and the supernatant was discarded when the proportion of cytopathic lesions (CPE) reached about 80% (about 12 h) (Figure 1B), and the cells were collected, frozen in liquid nitrogen, and kept in a −80 °C ultra-low freezer. Each group consisted of two biological replicates.

### 2.2. Library Construction and Sequencing

Total RNA was extracted from the samples, and the purity, concentration, and integrity of the RNA samples were determined by using a Nanodrop, Qubit 2.0, and Agilent 2100 methods to ensure that the samples to be used for transcriptional sequencing were of good quality. mRNA from the extracted total RNA was enriched and purified by using poly(T)-rich low-adsorption magnetic beads. A fragmentation buffer was added to randomly break the mRNA. Using mRNA as template, the first cDNA strand was synthesized using random hexamers. Then, the second cDNA strand was synthesized by adding a buffer, dNTPs, RNase H, and DNA polymerase I. The cDNA was purified by using AMPURE XP beads. The purified double-stranded cDNA was further subjected to terminal repair with A-tails and ligated into sequencing adapters. The AMPure XP beads were then used to select the fragment size. Finally, the cDNA library was obtained by PCR amplification. The established library was sequenced on an Illumina HiSeqTM 4000 system (San Diego, CA, USA).

### 2.3. Transcriptome Analysis

The raw data obtained by sequencing was initially processed. The data with a ratio of reads containing connectors and N (undetermined bases) >10%, along with the low-quality reads were discarded, and the corresponding clean reads database was obtained. The clean data of each sample were aligned and compared to the assembled unigene library. The reads that matched Unigenes were designated as mapped reads, which were then used in the subsequent analysis. The expression level of each gene in the sample was calculated using the reads per kb per monthly reads (RPKM) method [5] using only the number of reads and the total number of reads that could be matched to the reference sequence.

### 2.4. Data Assembly and Annotation

The clean data obtained after quality control was assembled using Trinity [12] (https://github.com/trinityrnaseq/trinityrnaseq/wiki) to produce unigenes [13]. Unigene sequences were aligned and compared to the NR [14], Swiss-Prot [15], GO [16], COG [17], KOG [18], eggNOG4.5 [19] and KEGG [20] databases by using BLAST [21]. KOBAS2.0 [22] was employed for KEGG analysis of Unigenes. The amino acid sequence of each unigene was predicted and then annotated based on the results of alignment and comparison using HMMER [23] and the Pfam [24] database.

### 2.5. Screening of Differentially Expressed Genes (DEGs)

The relative expression level of unigenes was determined by using the number of reads per kilobase of the map to the number of reads per 1 million base of the exon (FPKM). DEGs between the experimental and control groups were calculated using the DESeq [25] technique [26]. For differential expression analysis, the well-known Benjamin-Hochberg method was used to correct the p-value from the original test. Using the corrected p-value, the false discovery rate (FDR) was selected as the key index for screening DEGs. In this study, the FDR was <0.01, and the fold-change was ≥3.

### 2.6. Functional Annotation and Enrichment Analysis of DEGs

Gene Ontology (GO) is a functional annotation of the DEGs. Using the Goatools software (https://github.com/tanghaibao/GOatool) for feature enrichment, significant enrichment was observed when the FDR was <0.05. COG classification of DEGs from various groups of samples was also performed. KEGG pathway analysis using the KEGG Orthology-Based Annotation System (KOBAS; http://kobas.cbi.pku.edu.cn/home.do) was also conducted, and the signaling pathways were verified using the Fisher’s test and an FDR of ≤0.05.

### 2.7. SSR and SNP Identification

The analysis of the unigene sequences using MIcroSAtellite (MISA) software (http://pgrc.ipk-gatersleben.de/misa/misa.html) identified six types of SSRs: mononucleotide, dinucleotide, trinucleotide, tetranucleotide, pentanucleotide, and hexanucleotide repeats. Single nucleotide polymorphisms (SNP) were analyzed using STAR [27] (https://github.com/alexdobin/STAR).

### 2.8. Real-time Polymerase Chain Reaction Analysis

To validate the data generated from Illumina HiSeqTM 4000 sequencing (San Diego, CA, USA), 10 DEGs were randomly selected for real-time quantitative PCR (qPCR) analysis. Primers were designed using a primer software (Table 1). The total RNA used for RT-PCR was the same as that for the transcriptome. A Bole (CFX96) PCR instrument was used for PCR detection with β-actin as internal reference. The reaction system (total volume: 25 µL) consisted of the following: 12.5 µL SYBR Premix Ex Taq II, 1 µL of the upstream and downstream primers, 2.5 µL of the cDNA template, and supplemented with dH_2_O to a final reaction volume of 25 µL. To confirm that only one PCR product was amplified, the PCR product was subjected to dissociation curve analysis at the end of each PCR reaction. The data were analyzed by using the CFX (Computational Fluid Dynamics X) software (version3.1, Hercules, CA, USA) package using Ct values (2^−ΔΔCt^ values) to analyze the expression levels of different genes.

## 3. Results and Analysis

### 3.1. RNA Sequencing and Transcriptome Sequence Assembly

In the present study, high-throughput sequencing of EPC cells (T03 and T04) infected with reovirus and uninfected EPC cells (T01 and T02) was performed via high-throughput transcriptional analysis and short sequence assembly analysis. The purity, concentration, and integrity of the RNA of the four samples were assessed by using Nanodrop, Qubit 2.0, and the Aglient 2100 system. The results showed that the concentration and total amount of the four samples were in accordance with the sequencing requirements. Four samples were sequenced using a HiSeqTM 4000 (San Diego, CA, USA) high-throughput sequencing platform. After removing the linker and primer sequences, filtering low-quality data, and performing sequencing quality control, read number and base number of each sample were obtained (Table 2). The analysis found that the percentages of Q30 bases were not >89.44% and GC Content were >47.19%, which indicated that the sequencing results were good and could generate good original data for the subsequent data assembly.

**Figure 2:**
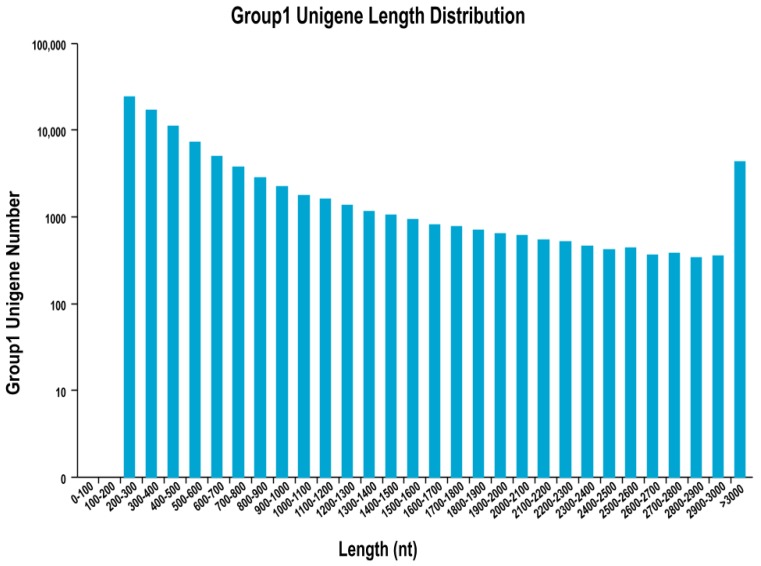
Unigene length distribution. The abscissa indicates the length of the unigene, and the ordinate indicates the number of unigene of a certain length.

Using Trinity (http://trinityrnaseq.sourceforge.net/) to assemble and splice the clean reads, the data was assembled into 92,101 contigs with an average length of 835.24 bp and a N50 value of 1432 nt. The length distribution of the reads is shown in Figure 2, with Unigene length 300–500 being the largest number, accounting for 30.23%, followed by Unigene length 200–300. The clean reads were mapped to Unigene, and the results are shown in Table 3, mapped ratio were >73.37%, and EPC cells (T03 and T04) infected with reovirus and uninfected EPC cells (T01 and T02) were similar, indicating better reproducibility. The assembly showed that the pattern of length distribution and average length of contigs were similar to the results of previous transcriptome studies using Illumina sequencing [28,29]. The large amount of sequence information obtained not only filled the gene expression profile of reovirus-immunized cyprinids, but also facilitated the mining of genetic information resources [30].

### 3.2. Functional Annotation and Classification of Transcriptional Group Data

The 92,101 assembled unigenes as describe above were compared to those in the NR, SWISS-PROT, eggNOG, COG, Pfam, KOG, GO, and KEGG databases respectively. By selecting the BLAST parameter E-value of < 1 × 10^−5^ and the HMMER parameter E-value of < 1 × 10^−10^, 42,522 unigene annotations were obtained. The results of the comparative analysis are shown in Table 4, compared to COG, CO, KEGG, KOG, Pfam, Swissport, eggNOG and NR in the database Unigene, respectively, 10,157 (23.9%), 20,555 (48.3%), 21,915 (51.5%), 27,599 (64.9%), 28,364 (66.7%), 22,535 (53%), 39,539 (92.9%), 41,260 (97%) Unigene. A total of 10,157 (23.9%) Unigene are annotated in at least one database.

### 3.3. Differential Gene Expression of EPC Cells Pre-infected and Post-infected by Reovirus from Allogynogenetic Crucian Carp

**Figure 3:**
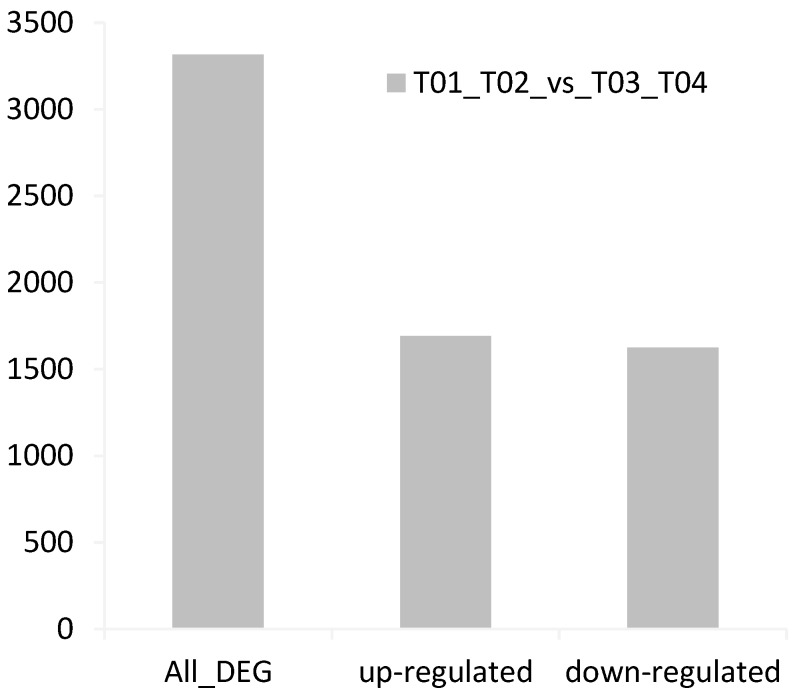
The number of differentially expressed genes. DEG Set: Name of differentially expressed gene set; All DEG: Number of differentially expressed genes; upregulated: TNumber of upregulated genes; downregulated: Number of downregulated genes.

**Figure 4:**
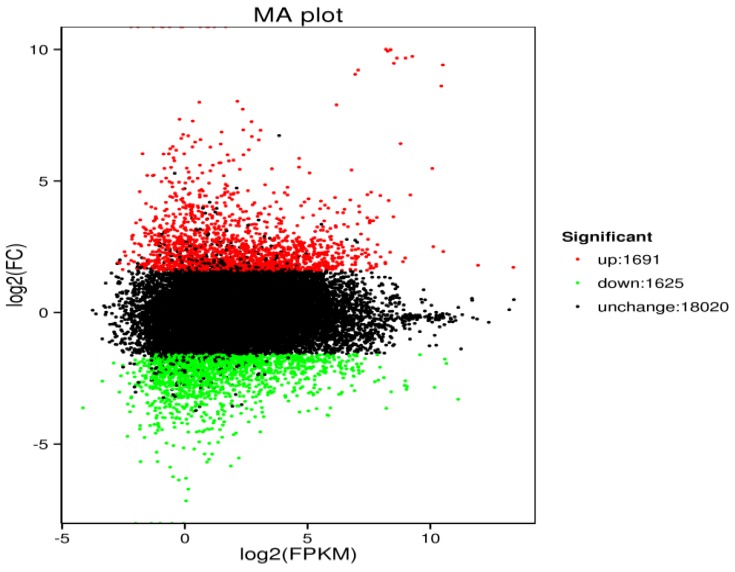
MA map of differentially expressed genes. Each point represents a gene in the MA map of differentially expressed genes. The abscissa is the A value: log2 (FPKM), the logarithm of the mean value of the expression in the two samples. The ordinate is the M value: log2 (FC), the logarithm of the difference in gene expression between the two samples, which was used to measure the difference in the level of expression. The green and red dots represent genes with significant differences in expression levels, green represents downregulated gene expression, red represents upregulated gene expression, and black spots represent genes with no significant differences in expression.

To identify DEGs that may play an important role in the defense response to reovirus infection in cyprinids, the data of the test groups (TO3 and T04) infected with reoviruses from allogynogenetic silver crucian carp were compared to that of the uninfected control groups (T01 and T02). A total of 3316 DEGs were identified by using the standard FDR of <0.01 and fold-change of ≥3, of which 1691 were upregulated and 1625 were downregulated (Figure 3). The distribution of DEGs is illustrated using an MA plot (Figure 4) [31]. To visualize the gene expression profiles, we generated a heatmap using the Hcluster algorithm, where the color changes from red to green with decreasing expression levels [32].

### 3.4. DEGs GO Functional Enrichment Analysis

**Figure 5:**
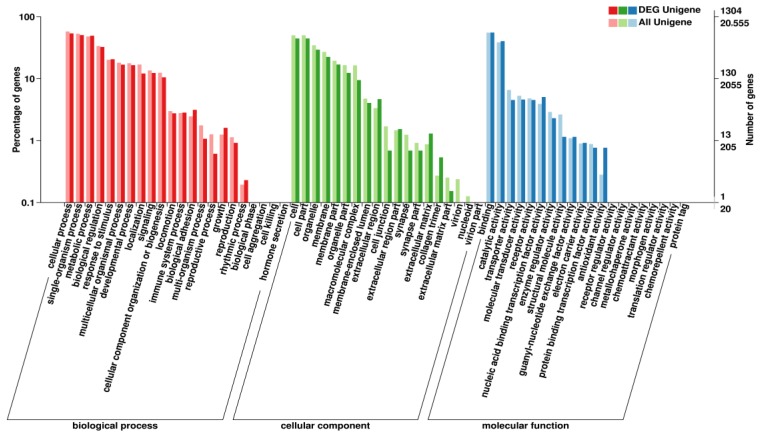
Clustering map of differentially expressed genes Gene Ontology (GO) in test group and control group. The abscissa is the GO classification, on the left of the ordinate is the percentage of the number of genes, and on the right is the number of genes. This figure shows the secondary function of each GO gene enrichment status in the background of the differentially expressed genes and the background of all the genes, thus reflecting the status of each secondary function in two backgrounds. The secondary function with significant difference indicates that the differentially expressed genes are different from the enrichment trend of all genes, and can be focused on whether this function is related to the difference.

The GO database is a structured standard biology annotation system constructed by the GO organization, which aims to establish a standard vocabulary system for genes and its products and is applicable to all species. To further reveal the function of DEGs, 3316 DEGs from the test group (T03 and T04) that were infected with allogynogenetic reovirus and the uninfected control group (T01 and T02) were subjected to GO Note (Figure 5). The results showed that 3941 GO terms were annotated, including 2719 biological processes (37,810 unigenes). Among the 376 cell components (7943 unigenes) and 846 molecular functions (11,750 unigenes), it is indicated that most of the differentially expressed genes are mainly involved in the biological process of cells, but there are few genes involved in cell components and molecular functions.The DEGs were mainly involved in cellular processes (GO: 0009987), single biological processes (GO: 0044699) (GO: 0044464), organelle (GO: 0043226), and catalytic activity (GO: 0003824). These results indicated that various physiological and biochemical changes occurred in the EPC cells during reovirus infection, and these played an important role in virus defense response, it is possible to recognize the functional distribution of differentially expressed genes from the macroscopic angle of infection of the infected reovirus.

### 3.5. COG Annotation of DEGs

**Figure 6:**
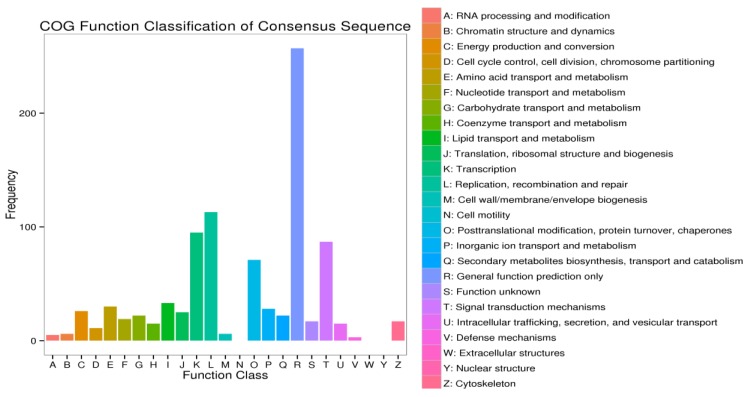
Orthologous classification of the differentially expressed genes in the COG (Clusters of Orthologous Groups) database between the experimental group and the control group. The abscissa is the COG (Clusters of Orthologous Groups) content of each classification, and the ordinate is the number of genes. In different functional categories, the number of genes reflects the corresponding period and the environment under the metabolic or physiological biases and other content, and the object can be combined with the distribution of various functional classes to generate a scientific explanation.

COG (Clusters of Orthologous Groups) is a database of orthologous classification of gene products. Each COG protein is presumed to be derived from an ancestral protein. The COG database was constructed based on bacterial, algal, eukaryotic encoded proteins with complete genomes and phylogenetic relationships. A total of 3166 differentially expressed genes from the comparative analysis of the test group (TO3 and T04) that was infected with allogynogenetic reovirus to the uninfected control groups (T01 and T02) were directly orthologous and classified with gene products in the COG database. The resulting classification is shown in Figure 6. A total of 25 different functions were involved. According to the number of genes arranged from high to low in the functional classification, the number of functional units in the first six positions were 257 in general function prediction (R), 113 in replication, recombination and repair (L), 71 in translational modification, protein turnover, chaperone (O), 33 in lipid transport and metabolism (I), 87 in signal transduction mechanism (T), 95 in transcription (K), 36 in each of secondary metabolite synthesis, transport and decomposition (Q), and amino acid transport and metabolism (E). It shows that EPC cells can resist the injury by adjusting their physical metabolism in the face of the reovirus infection.

### 3.6. Pathway Enrichment Analysis of DEGs

**Figure 7:**
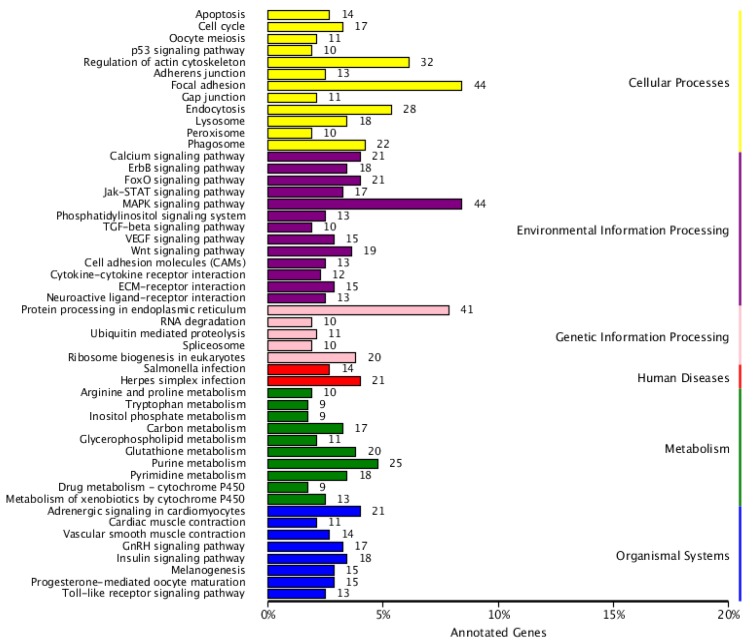
KEGG classification of differentially expressed genes. The ordinate is the name of the KEGG metabolic pathway, and the abscissa is the ratio of the number of genes annotated to the pathway and the number of genes in the annotated genes.

The execution of biological functions relies on the coordination among genes. Pathway enrichment analysis of DEGs could provide better insights into the biological function of a gene and its interaction with other genes, so as to further study their complex behavior. The differential genes in the test group (T03 and T04) infected with reovirus and the uninfected control group (T01 and T02) were found to be involved in 50 subclasses of metabolic pathways in six broad categories (cellular processes, environmental information processing, genetic information processing, human diseases, metabolism, and organic systems) after these were analyzed by using the metabolic pathway database KEGG (Figure 7). Among these, several signaling pathways with a relatively high number of genes were identified, which included actin cytoskeleton regulation (ko04810), adhesion (ko04510), MAPK signaling pathway (ko04010), and protein processing in the endoplasmic reticulum (ko04141). These metabolic pathways are involved in the signal transduction of environmental information processing. There were 35 pathways associated with immune and disease resistance, including cytokine-cytokine-receptor interaction (12 genes, ko04060), apoptotic signaling pathway (21 genes, ko04068), phagocytosis (22 genes, ko04145), endocytosis (28 genes, ko04144), and Toll receptor signaling pathway (13 genes, ko04620). These pathways are basically involved in all aspects of fish immunization. This demonstrates that the experimental KEGG annotation effect is good and shows the reovirus and immune-induced regulatory pathways. Genes and pathways associated with the immune system, signal transduction and disease processes were similar to those previously reported in zebrafish [33], grouper [34], and bream [35]. Studies on immune-related genes and pathways can improve our understanding of the molecular immune mechanisms of reovirus-stimulated EPCs.

### 3.7. SSR Tags

Among various molecular markers, SSRs have many recognized functions and are widely used in paternity testing, genetic diversity, some aspects of genetic linkage maps, and marker-assisted breeding [36]. SSRs are tandem repeats of single nucleotides to hexanucleotides found in all prokaryotic and eukaryotic genomes. As a molecular marker, SSRs have a variety of putative functions and high polymorphisms that play an important role in certain aspects of genetic and breeding research. In addition, gene transcription and mRNA splicing or export to the cytoplasm may be affected by intron SSRs. Therefore, it is essential to understand the spectrum of SSRs. In the present study, we determined that all 21,985 unigenes in the transcriptome group contained SSRs (Table 5).

SNPs are the most abundant type of DNA sequence polymorphisms and have been increasingly used in quantitative trait loci (QTLs) for molecular marker linkage map construction, mapping, and association studies [37,38]. According to the number of alleles (Allele) on the SNP locus, i.e., the number of different bases supported by the sequencing reads, SNP sites can be divided into homozygous SNP sites (only one allele) and heterozygous SNP sites (two or more alleles). The percentage of heterozygous SNPs in different species varies. The number of SNP sites in each sample in this study is shown in Table 6. Both the Homo SNP and HeteSNP crucian carp were less in the test group (T03 and T04) infected with reovirus and the uninfected control group (T01 and T02). We can further study genetic markers related to immune-related genes and provide reference for the pathogenesis of reovirus-infected cyprinid fish and the search for genetic markers of immune genes.

### 3.8. Real-Time PCR Verification of DEGs

**Figure 8:**
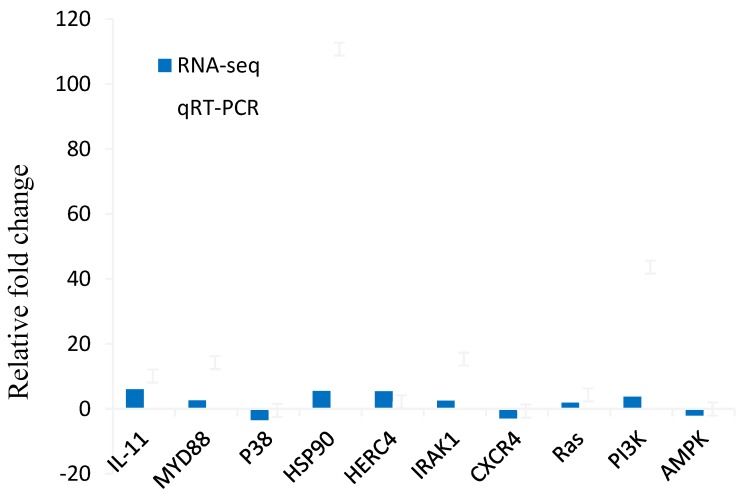
Comparison of relative fold changes between RNA-seq and qRT-PCR results.

The selected genes were associated with cytokine receptors, Toll-like receptor signaling conduction (TLR), chemokine signaling pathways, and MAPK signaling pathways. Compared to the control group, the expression level of these genes in the EPC cells infected with reoviruses was consistent with that generated from transcriptome analysis (Figure 8).

## 4. Discussion

The molecular response mechanisms of animals following viral infection include changes in transcriptional and translational levels, which in turn regulate different metabolic pathways and signaling pathways. Therefore, transcriptome research has become an important direction of functional genomics research. The second-generation transcriptome sequencing (RNA-Seq) technology is a high-throughput sequencing approach involving the cDNA from fragmentally processed mRNA, sequence splicing assembly, and statistical correlation of number of sequences to obtain different transcripts (mRNA). This technique has been widely applied to the determination and analysis of host-virus interaction transcriptome. So far, studies on the mechanisms underlying the molecular immune response of cyprinids to reoviruses on cyprinids are limited. To fully understand the molecular mechanism underlying reovirus response, we first analyzed the transcriptome data of EPC cells before and after reovirus infection as well as obtained gene data. We then analyzed various gene expression profiles to identify genes that are involved in the immune response and signal transduction (Table 7).

**Figure 9:**
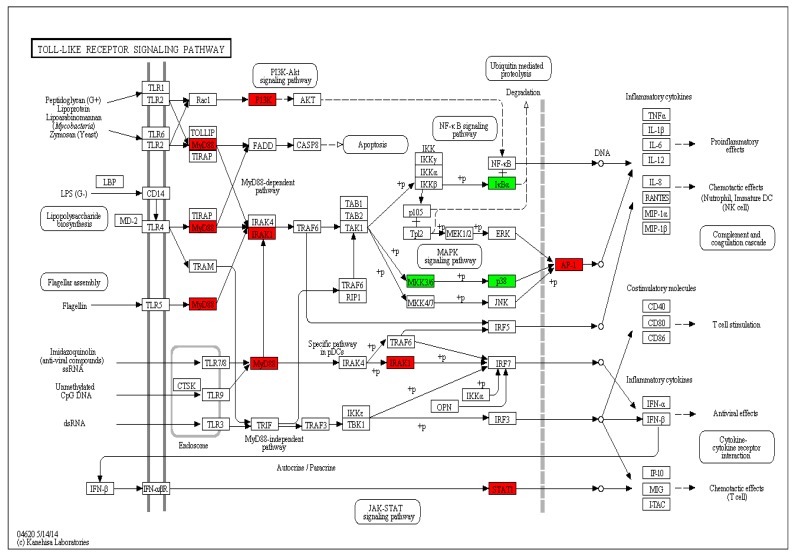
Significantly differentially expressed genes identified by KEGG that are involved in Toll-like receptor signaling pathway. Red boxes indicate upregulated DEGs; Green boxes represent downregulated DEGs; (For interpretation of colors used in this figure, please refer to the web version of this article).

The immune system of fish provides protection from the pathogens, thereby allowing its survival and evolution. The Toll-like receptor family that mediates immune congenital immunity is the first line of defense of fish and plays a role in connecting innate immunity and acquired immune responses. Thirteen representative immune-related genes were significantly differentially expressed in the Toll-like receptor signaling pathway (Table 7, Figure 9). The MyD88 gene was significantly upregulated in the metabolic pathways of EPC cells after infection with reovirus, suggesting that it might play a key role in the generation of anti-reoviruses in carp and thus conferring pathogen resistance, which is similar to the findings of previous research studies [39,40,41,42]. In addition, the expression of the PIK3C and TAK1 genes were significantly downregulated after reovirus stimulation, which may be a negative regulatory mechanism of excessive inflammatory response. The results of this study differed from those of other viral infections, suggesting that certain genes in the Toll-like receptor signaling pathway play a key role in the response to reoviruses. Up-regulated expression of AP-1 and STAT after reovirus infection can promote the expression of proinflammatory cytokines and inflammatory cytokines, such as TNFα, IL-12/IL-1β. The release of these cytokines extracellularly can cause cells, macrophages chemotactic aggregation, increased capillary permeability, lymphocyte infiltration, and other inflammatory reactions.

**Figure 10:**
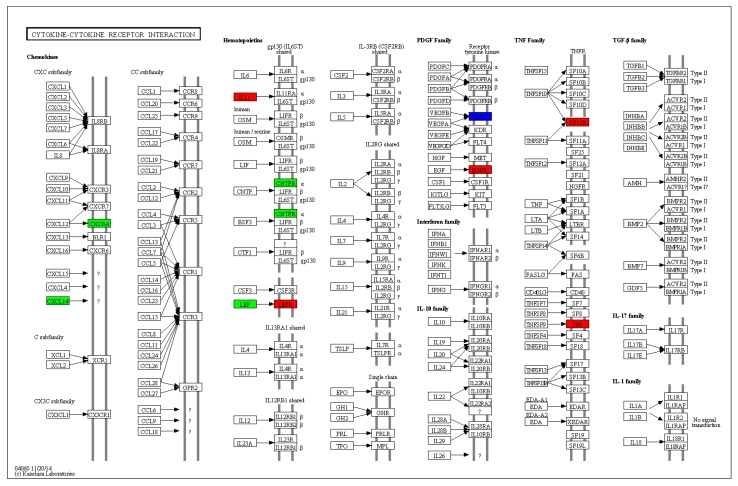
Significantly differentially expressed genes identified by KEGG that are involved in the cytokine-cytokine receptor interaction signaling pathway. Red boxes indicate upregulated DEGs; Green boxes represent downregulated DEGs; Blue boxes show no changes in expression. (For interpretation of colors used in this figure, please refer to the web version of this article).

The interaction between cytokines and membrane receptors is an important link in various biological activities. Cancer, autoimmune diseases, metabolic disorders, and other diseases are closely related to cytokine imbalance, and studying the interactions between cytokines and receptors becomes the cornerstone for the development of treatment of these diseases. Cytokines are a class of high activity, multi-functional protein peptide molecules or glycoproteins that are produced by activated immune cells (e.g., lymphocytes and mononuclear macrophages) and related cells (e.g., fibroblasts and endothelial cells) and thereby regulate cell function. These interact with specific receptors with high affinity, and are an indispensable component of the immune system. Approximately 12 representative immune-related genes were significantly differentially expressed in cytokine-cytokine receptor interaction signaling pathways after being infected with reovirus (Table 7, Figure 10). CXCL14 is a novel chemokine that decreases the inflammatory response. The CXCL14 gene was significantly downregulated in the metabolic pathways of EPC cells after being infected with reoviruses, thereby indicating that the inflammatory reaction may play a role in preventing the development of reoviruses. CXCR4 plays an important role in immunity, organogenesis and repair, anti-cancer, embryonic development, and hematopoiesis. CXCR4 can prevent the occurrence of excessive inflammatory reaction by negatively regulating the production of immune factors. In the present study, the CXCR4 gene was significantly downregulated in the metabolic pathways of EPC cells after infection with reovirus, indicating that it plays an important role in resistance against reovirus infection. The study of cytokine gene expression regulation may identify antagonists or activators that actively adjust the expression of specific genes. In addition, these studies may facilitate the establishment of an efficient eukaryotic expression system that generated a high yield of recombinant cytokines for the treatment of specific diseases or vaccine adjuvants.

**Figure 11:**
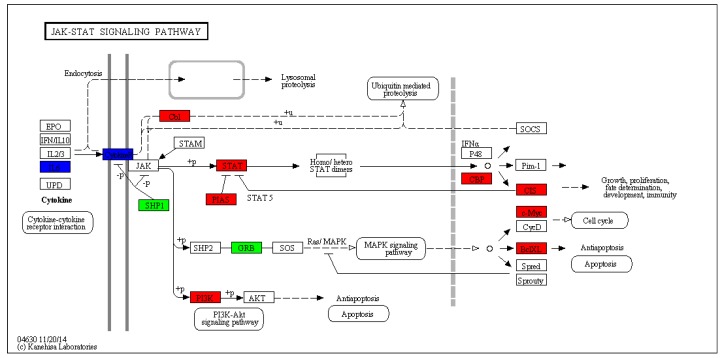
Significantly differentially expressed genes identified by KEGG that are involved in the Jak-STAT signaling pathway. Red boxes indicate upregulated DEGs; Green boxes represented downregulated DEGs; Blue boxes show no changes in expression. (For interpretation of colors used in this figure, please refer to the web version of this article).

The JAK-STAT signaling pathway mediates cytokine signal transduction. It is involved in multiple life processes such as disease infection, immunity, inflammation, and development. In this study, ten representative immune-related genes were significantly differentially expressed in the JAK-STAT signaling pathway (Table 6, Figure 11). The three main members of the JAK-STAT signaling pathway are cytokine receptors, JAK, and STAT. STAT is a unique protein family that binds to DNA. So far, seven members of the STAT family have been identified, which include STAT1, STAT2, STAT3, STAT4, STAT5a, STAT5b, and STAT6. STAT1 is mainly involved in the response to IFNs, STAT1 deficiency can cause increased susceptibility to viral infection and disorders of intracellular pathogen infection regulation. In the present study, cytokines, growth factors, and other factors activated JAK, and then activated STAT1 after binding to their associated receptors. The STAT1 and CISH (cytokine inducible SH2-containing protein) were significantly upregulated in EPC cells that were infected with reoviruses, indicating that their susceptibility to virus infection decreased, the body was stimulated to activate the immune system, and the disorders of intracellular pathogen infection regulation were reduced. The role of IL-6 and receptor genes in the JAK-STAT signaling pathway may be further investigated. The PIK3R gene was upregulated in EPC cells after reovirus infection, possibly promoting apoptosis. This study laid the foundation for further investigating the role of the fish JAK-STAT gene in fish immune regulation and disease control. By performing series of analyses and research studies on the JAK-STAT signaling pathway, schemes for disease control and targets for the development of new drugs may be established.

**Figure 12:**
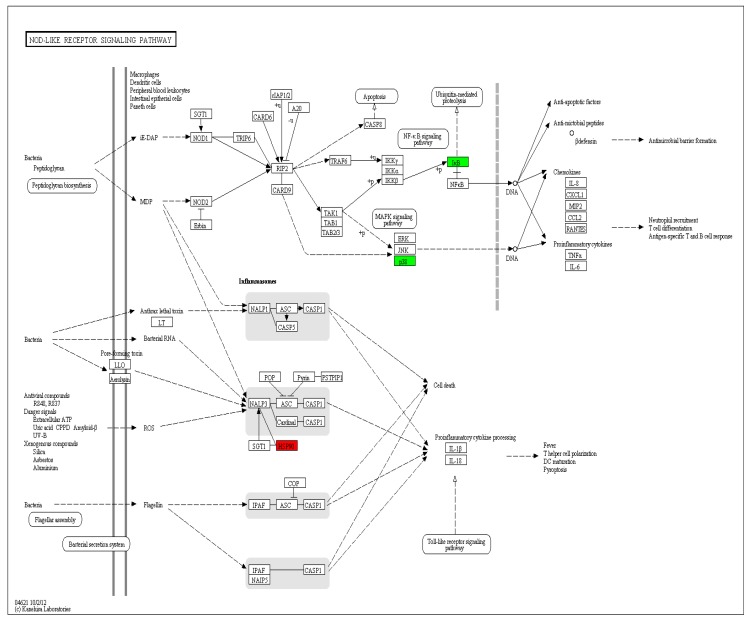
Significantly differentially expressed genes identified by KEGG that are involved in the NOD-like receptor signaling pathway. Red boxes indicate upregulated DEGs; Green boxes represented downregulated DEGs; (For interpretation of colors used in this figure, please refer to the web version of this article).

NLRs were found recently, they are cytoplasmic pattern recognition receptors (PRRS). NLRs play an important role in defending against pathogen infection by recognizing pathogen-associated molecular patterns (PAMPs) and initiating the innate immune response, leading to an adaptive immune response through signal transduction. Approximately 3 representative immune-related genes were significantly differentially expressed in NOD-like receptor signaling pathway after being infected with reovirus (Figure 12). Among them, the significant down-regulation of IkB and P38 inhibits NOD1 (Nucleotide-binding oligomerization domain-containing protein 1) directly recruiting RIPK2 through CARD-CARD (Caspase activation and recruitment domains) interaction during viral recognition, further activating NF-kB and MAPK (mitogen-activated protein kinase) signaling and stimulating the body to produce immunity. In this study, there was no difference in RIPK2, which may be due to the early stage of Infection increased and then decreased in the early stage of Infection increased and then decreased, Or the amount of expression increased but the difference was not significant compared with the control group, which can be further studied in the follow-up. It has been demonstrated that the HSP90 and SGT1 interact with the LRR (leucine-rich repeat) domain of NLRP3 (leucine-rich repeat and pyrin domain domains-containing protein 3) to maintain a non-activated but stable state, activating NLRP3 inflammation through changes in HSP90 The body causes secretion of IL-1β or IL-18 as well as programmed cell death. In addition HSP90 involved in a variety of physiological processes in vivo, as a molecular chaperone to improve the heat resistance of organisms and protect organisms and other functions. The infection of reovirus HSP90 in EPC cells significantly increased, indicating that not only by regulating its receptor protein activity and maintaining the stability of the receptor protein structure and indirectly involved in the regulation of multiple intracellular signaling pathways, but also to deal with resistance pathogen invasion and regulation of the body’s immune system also plays an important role, suggesting that it might play a key role in the generation of anti-reoviruses in carp and thus conferring pathogen resistance, which is similar to the findings of previous research studies [43,44].

In addition to the Toll-like and JAK-STAT signaling pathways that interact with cytokines and cytokine receptors, various representative immune system-related genes are significantly expressed in phagocytosis and chemokine- and apoptosis-related pathways. Some genes play an important role in other pathways such as Ras; for example, it is involved in the regulation of the actin cytoskeleton, the MAPK signal transduction pathway, and the chemokine pathway; Furthermore, MyD88 is involved in apoptosis and the Toll pathway. The identification of these common genes in different pathways may improve our understanding of the molecular immune mechanisms of reovirus-infected cyprinids.

## 5. Conclusions

In the present study, comparative transcriptome analysis of EPC cells infected with reovirus and uninfected EPC cells was performed to explore the molecular mechanisms of immunity and the development of molecular markers. EPC cells infected with reoviruses showed significant changes in the expression of a variety of immune-related pathways such as Toll-like receptor signaling, cytokine-cytokine receptor interactions, chemokine signaling pathways, and endocytosis. In addition, the identification of 21,985 SSR markers and 62,992 SNPs in the transcriptome of EPC cells facilitated the development of markers, the analysis of inheritance patterns, and establishment of QTLs. In summary, this study provides insights into the immunoprophylaxis of reovirus-infected cyprinids and provides a theoretical basis for the pathogenesis and immunological pathways of Cypriniform reoviruses, as well as providing useful information for fish farming.

## Figures and Tables

**Table 1:** Primers, sequences, target genes, product sizes, and applications used in the present study.

Name	Sequence (5′ → 3′)	Product Size (bp)
IL-11F	TAGAGCACTGCTAGGCCTGA	124
IL-11R	TACCACGATGCCTGTTGACC	
MYD88F	GAGGACAGTCGCCGAAATGA	123
MYD88R	TTTGCTACAGTGGCCTCTGG	
P38F	CTTTCTTCCCACTCGCTGGT	146
P38R	AATCCCTCTGCTTGTCTGCC	
HSP90F	ATGGTGGAGTTGTCTCGCAG	127
HSP90R	TTGAGAAGGTCACCGTGTCG	
HERC4F	ACAGGTGGAGGGCTTAGGTT	133
HERC4R	CGGTGTGTTGGAAAACCTCT	
IRAK1F	GGCATCCCAACATAATGGAC	119
IRAK1R	GTTGCTGTCCTCACAGCGTA	
CXCR4F	ACCGTCATCCTCATCCTTTG	86
CXCR4R	ACGTTCAGCTCCACCAGAGT	
RasF	ACGACCCAACCATTGAGGAC	131
RasR	TCCGGTCCTCATGTACTGGT	
PI3KF	GATGGAGCCTTCCTCATCCG	135
PI3KR	CAAGTTGTAGGGCTCTGCGA	
AMPKF	TACCGTGCCATGAAACAGCT	130
AMPKR	GGTTGTCCACCTGGTAGAGC	
β-actinF	TGCAGTCAACATCTGGAATC	191
β-actinR	ATTTTTGGCGCTTGACTCAG	

**Table 2:** Statistical data for sample sequencing.

Sample	Read Number	Base Number	GC Content	% ≥Q30
T01	36,262,497	10,633,672,216	47.49%	89.44%
T02	28,349,442	8,398,124,460	47.15%	89.57%
T03	28,251,699	8,299,768,760	48.54%	89.91%
T04	29,010,341	8,549,124,730	47.87%	90.09%

Note: Samples: Sample name on the sample information sheet; Read number: Total number of paired-end reads in the clean data; Base number: Total number of bases in the clean data; GC content: GC content in the clean data, which is the percentage of G and C bases in the total bases in the clean data; % ≥Q30: Percentage of bases with a mass value ≥ 30 in the clean data.

**Table 3:** Comparison of sequencing data and assembly results.

Sample	Number of Clean Reads	Number of Mapped Reads	Mapped Ratio
T01	36,262,497	26,659,090	73.51%
T02	28,349,442	20,801,516	73.37%
T03	28,251,699	21,018,859	74.39%
T04	29,010,341	21,523,297	74.19%

Note: Samples: Sample name on the sample information sheet; Clean reads: Number of clean reads, paired end; Mapped reads: Number of mapped reads, paired end; Mapped ratio: Percentage ratio of mapped reads in the clean reads.

**Table 4:** Distribution of annotated unigenes.

Anno_Database	Annotated_Number	300 ≤ Length < 1000	Length ≥ 1000
COG_Annotation	10,157	2887	6538
GO_Annotation	20,555	6433	12,164
KEGG_Annotation	21,915	7875	11,248
KOG_Annotation	27,599	9471	14,856
Pfam_Annotation	28,364	8584	17,552
Swissprot_Annotation	22,535	7054	13,358
eggNOG_Annotation	39,539	14,403	19,799
nr_Annotation	41,260	15,294	20,454
All_Annotated	42,522	15,898	20,572

Note: Annotated databases: the function of the database; Annotated_Number: Number of comments annotated to the unigene database; 300 ≤ Length < 1000: Number of unigenes annotated to the database that is ≤300 or less and <1000 bases in length; Length ≥ 1000: Number of unigenes annotated to the database that are >1000 bases in length.

**Table 5:** Statistical table of SSR analysis results.

#Type	Number
c	1556
c *	68
p1	11,943
p2	6079
p3	2078
p4	249
p5	10
p6	2
Total	21,985

Note: Total number of identified sequences: Total number of sequences evaluated: Total size of examined sequences (bp): Number of total bases evaluated; Total number of identified SSRs: Total number of SSRs identified; Number of SSR containing sequences: number of sequences containing SSRs; Number of sequences containing >1 SSR: Number of sequences containing >1 SSR and SSR overlaps each other; Number of SSRs present in compound formation: Number of SSRs in complex form; Mononucleotide: Single-base repeat SSR; Dinucleotide: Double-base repeat SSR; Trinucleotide: Three-base repeat SSR; Tetranucleotide: Four-base repeat SSR; Pentanucleotide: Five-base repeat SSR; Hexanucleotide: Six-base repeat SSR.

**Table 6:** SNP distribution.

Sample	HomoSNP	HeteSNP	AllSNP
T01	37,230	66,496	103,726
T02	35,852	60,150	96,002
T03	35,445	57,150	92,595
T04	34,355	53,879	88,234

**Table 7:** Representative immune-related genes that are differentially expressed after reovirus infection.

Gene Name	Description	Change	log2FC	FDR
T-cell receptor signaling pathway				
Ras	GTPase HRas	Up	1.84	0.000154
SNARE interactions in vesicular transport				
VAMP4	Vesicle-associated membrane protein 4	Up	1.68	0.005729
Nicotinate and nicotinamide metabolism				
PNP	Purine-nucleoside phosphorylase	Up	1.92	4.45 × 10^−17^
NRK1_2	Nicotinamide/nicotinate riboside kinase	Up	1.61	4.37 × 10^−6^
Phagosome				
ATPeV0A	V-type H+-transporting ATPase subunit a	Up	1.67	6.01 × 10^−9^
TUBB	Tubulin beta	Up	2.17	1.48 × 10^−6^
C1R	Complement component 1, r subcomponent	Down	−2.39	5.62 × 10^−6^
ITGB2	Integrin beta 2	Down	−1.73	5.78 × 10^−5^
MRC	Mannose receptor, C type	Up	2.91	2.73 × 10^−8^
Jak-STAT signaling pathway				
STAT1	Signal transducer and activator of transcription 1	Up	1.62	6.02 × 10^−5^
CBL	E3 ubiquitin-protein ligase CBL	Up	2.28	0.007492469
SHP-1	Tyrosine-protein phosphatase non-receptor type 6	Down	−2.54	5.79 × 10^−7^
GRB2	Growth factor receptor-binding protein 2	Down	−1.74	5.42 × 10^−5^
EP300	E1A/CREB-binding protein	Up	1.67	0.000264763
CISH	Cytokine-inducible SH2-containing protein	Up	3.38	4.58 × 10^−30^
MYC	Myc proto-oncogene protein	Up	3.49	9.21 × 10^−29^
BCL2L1	Bcl-2-like 1 (apoptosis regulator Bcl-X)	Up	2.19	0.000500092
Toll-like receptor signaling pathway				
PIK3C	Phosphatidylinositol-4,5-bisphosphate 3-kinase	Up	3.73	7.41 × 10^−32^
MYD88	Myeloid differentiation primary response protein MyD88	Up	2.61	1.96 × 10^−11^
IRAK1	Interleukin-1 receptor-associated kinase 1	Up	2.49	1.63 × 10^−22^
NFKBIA	NF-kappa-B inhibitor alpha	Down	−3.11	4.49 × 10^−17^
MAP2K6	Mitogen-activated protein kinase 6	Down	−3.60	1.29 × 10^−8^
P38	p38 MAP kinase	Down	−3.04	5.50 × 10^−10^
FOS	Proto-oncogene protein c-fos	Up	2.30	6.13 × 10^−6^
Cytokine-cytokine receptor interaction				
CXCR4	C-X-C chemokine receptor type 4	Down	−2.93	4.62 × 10^−7^
CXCL14	C-X-C motif chemokine 14	Down	−1.65	1.04 × 10^−12^
IL11	Interleukin 11	Up	6.03	0.00595422
CNTFR	Ciliary neurotrophic factor receptor	Down	−1.86	0.001965213
LEP	Leptin	Down	−4.19	1.23 × 10^−7^
LEPR	Leptin receptor	Up	2.54	1.02 × 10^−7^
EGFR	Epidermal growth factor receptor	Up	2.22	5.09 × 10^−5^
TNFRSF11B	Tumor necrosis factor receptor superfamily member 11B	Up	4.86	9.75 × 10^−40^
TNFRSF9	Tumor necrosis factor receptor superfamily member 9	Up	4.37	7.65 × 10^−15^
Apoptosis				
TNFRSF10	Tumor necrosis factor receptor superfamily member 10	Up	2.26	0.000275944
MYD88	Myeloid differentiation primary response protein MyD88	Up	2.26	0.000275944
IRAK1	Interleukin-1 receptor-associated kinase 1	Up	2.49	1.63 × 10^−22^
MAP3K14	Mitogen-activated protein kinase 14	Up	3.13	6.78 × 10^−8^
PIK3C	Phosphatidylinositol-4,5-bisphosphate 3-kinase	UP	3.73	7.41 × 10^−32^
CAPN2	Calpain-2	Down	−1.60	6.44 × 10^−9^
CFLAR	CASP8 and FADD-like apoptosis regulator	Up	2.14	2.40 × 10^−5^
BCL2L1	Bcl-2-like 1 (apoptosis regulator Bcl-X)	Up	2.19	0.000500092
NFKBIA	NF-kappa-B inhibitor alpha	Down	−3.11	4.49 × 10^−17^
XIAP	E3 ubiquitin-protein ligase XIAP	Up	1.60	3.86 × 10^−12^
CASP3	Caspase 3	Down	−2.28	1.94 × 10^−14^
CASP9	Caspase 9	Up	2.20	9.13 × 10^−15^
mTOR signaling pathway				
PIK3R	Phosphoinositide-3-kinase, regulatory subunit	Up	3.73	7.41 × 10^−32^
PRKCA	Classical protein kinase C alpha type	Down	−1.69	1.46 × 10^−7^
AKT1S1	Proline-rich AKT1 substrate 1	Down	−1.63	4.59 × 10^−7^
AMPK	5′-AMP-activated protein kinase, catalytic alpha subunit	Down	−2.06	0.000638513
